# Supplementary material for: Identification and functional analysis of non-coding regulatory small RNA FenSr3 in Bacillus amyloliquefaciens LPB-18
Source: PeerJ. 2023 May 15;11:e15236. doi: 10.7717/peerj.15236 (PMC10194069; doi:10.7717/peerj.15236)
Supplement: Supplemental Information 4 [file peerj-11-15236-s004.zip › KO/CK-vs-T1_map/map05412.html]

KEGG PATHWAY: Arrhythmogenic right ventricular cardiomyopathy (ARVC) - Reference pathway


|  |  |
| --- | --- |
| **Arrhythmogenic right ventricular cardiomyopathy (ARVC) - Reference pathway** |  |

[
Pathway menu
| Organism menu
| Pathway entry
| Show description
| User data mapping
]

Arrhythmogenic right ventricular cardiomyopathy (ARVC) is an inherited heart muscle disease that may result in arrhythmia, heart failure, and sudden death. The hallmark pathological findings are progressive myocyte loss and fibrofatty replacement, with a predilection for the right ventricle. A number of genetic studies have identified mutations in various components of the cardiac desmosome that have important roles in the pathogenesis of ARVC. Disruption of desmosomal function by defective proteins might lead to death of myocytes under mechanical stress. The myocardial injury may be accompanied by inflammation. Since regeneration of cardiac myocytes is limited, repair by fibrofatty replacement occurs. Several studies have implicated that desmosome dysfunction results in the delocalization and nuclear translocation of plakoglobin. As a result, competition between plakoglobin and beta-catenin will lead to the inhibition of Wnt/beta-catenin signaling, resulting in a shift from a myocyte fate towards an adipocyte fate of cells. The ryanodine receptor plays a crucial part in electromechanical coupling by control of release of calcium from the sarcoplasmic reticulum into the cytosol. Therefore, defects in this receptor could result in an imbalance of calcium homeostasis that might trigger cell death.

|  |  |  |
| --- | --- | --- |
| Reference pathway | 184% 150% 122% 100% 82% 67% 55% | 图片下载 |
